# Time interval between (chemo)radiotherapy and subsequent laryngectomy is not prognostic for post operative complications and survival

**DOI:** 10.1007/s00405-020-06384-y

**Published:** 2020-09-29

**Authors:** Thomas F. Pézier, Johannes A. Rijken, Bernard M. Tijink, W. Weibel Braunius, Remco de Bree

**Affiliations:** Department of Head and Neck Surgical Oncology, University Medical Center Utrecht, Utrecht, The Netherlands

**Keywords:** Pharyngo-cutaneous fistula, Laryngeal cancer, Total laryngectomy, Radiotherapy, Chemoradiation

## Abstract

**Purpose:**

Pharyngocutaneous fistula (PCF) formation and swallowing difficulties are common and troublesome complications following total laryngectomy (TL). Prior (chemo)radiotherapy ((C)RT) is thought to be a risk factor for these complications, but there is conflicting evidence as to whether the time interval between (C)RT and TL is important. The impact of time interval on these complications and also its impact on overall survival are investigated.

**Methods:**

This is a retrospective case note review of all patients undergoing TL at the University Medical Center, Utrecht, The Netherlands over the 10-year period from January 2008 to December 2017. The cohort was split into those who underwent TL within a year of finishing (C)RT and those longer than 1 year.

**Results:**

One hundred and twenty-six patients (108 males, 18 females), with a mean age of 66 underwent total laryngectomy after prior (C)RT in the study period. Overall 5-year survival was 35% with a median follow-up of 30 months. Fifty-four patients underwent laryngectomy within a year of their (C)RT versus 72 patients who had a time interval of more than one year. No differences in PCF rate, risk of dilatation or overall survival could be found between the two groups.

**Conclusions:**

In this modern cohort, time interval between (C)RT and surgery did not impact PCF rate, risk of dilatation or overall survival.

## Introduction

NCCN guidelines for the treatment of advanced stage laryngeal cancer [1] include laryngectomy, (chemo)radiotherapy or some combination thereof. Following the move towards organ-sparing approaches driven by the VA [2], RTOG and other studies [3, 4], surgeons are increasingly performing total laryngectomy as a salvage procedure following failed (chemo)radiotherapy ((C)RT), or as functional treatment for recurrent aspiration and airway issues following successful (C)RT setting. Indeed, for many units, there is now an almost 50/50 split between primary patients and patients who have undergone prior radiotherapy. This latter group can be split into patients with or without cancer and, therefore, includes patients undergoing salvage laryngectomy and a proportion of patients undergoing functional laryngectomy.

From our knowledge of radio-biochemistry, we know that radiotherapy is toxic to both cancerous and healthy cells. An initial acute inflammation lasts 2–3 months and includes endothelial injury resulting in a reduction in the size of the capillary bed [5]. Chronic changes include increased subintimal fibrosis, loss of vascular smooth muscle and endarteritis obliterans leading to fragile hypovascular, hypocellular and hypoxic tissue [6], a situation which generally stabilizes after 12–18 months.

These changes have several implications for post-radiotherapy management. Perhaps, the best investigated is the timing of imaging. For example, the timing of response evaluation by PET/CT has been extensively studied with a consensus that this is best performed after 3 months, i.e. after the initial acute inflammatory phase [7–10]. Another implication for post-radiotherapy management concerns the timing of surgery. Whilst some surgeries are strongly indicated (e.g. recurrent cancer), others may be relatively indicated (e.g. stricture release) and the timing of especially these latter operations can be informed by our understanding of the tissue toxicity. The timing of neck dissections following (C)RT has also been well studied. Stenson et al. [11] suggests a safe window of surgery somewhere between 4 and 12 weeks, though a more recent study comparing complications between groups undergoing neck dissection less than 12 weeks or more than 12 weeks and more showed no difference in overall complication rates [12].

Total laryngectomy is, however, an order of magnitude more invasive than a neck dissection with implications for speech [13], swallow [14] and breathing. It is associated with high levels of complications, particularly of pharyngo-cutaneous fistula (PCF) formation [15] with published results with rates of up to 58% in patients after prior (chemo)radiotherapy [16]. Several authors have highlighted the time interval between radiotherapy and subsequent laryngectomy as being a risk factor for PCF [17, 18] whilst others find no effect [19, 20]. One even finds a longer time interval as a risk factor for PCF [21]. Various cut-offs are used to describe a short vs long interval and few papers report overall survival outcomes or other complications such as swallowing difficulties needing dilatation.

This paper investigates the impact of time interval between primary (chemo)radiotherapy and laryngectomy with regards to PCF, risk of dilatation and overall survival.

## Materials and methods

A retrospective cohort study of all patients undergoing total laryngectomy following prior (chemo)radiotherapy at the University Medical Center, Utrecht over the 10-year period, Jan 2008–Dec 2017, was performed. The indication for total laryngectomy was either as salvage treatment for residual or recurrent cancer following (chemo)radiotherapy or as a treatment for a dysfunctional larynx following successful (chemo)radiotherapy.

Patients’ demographic, staging, treatment and outcome data were collected using electronic patient records. TNM classification according to the then applicable AJCC manual was recorded. All patients were discussed in our multidisciplinary tumor board and underwent total laryngectomy with or without (partial) pharyngectomy. For patients where the pharynx could not be closed primarily, a variety of flaps were used including pectoralis major with or without skin island, radial forearm free flap (RFFF), anterior lateral thigh flap (ALT), jejunum interposition and gastric pull-up. We did not make routine use of salivary bypass tubes during laryngectomy, preferring to use them only if patients developed a PCF.

Patients were stratified by time interval between end of (chemo)radiotherapy and date of laryngectomy. As per Basheeth et al. [18], a cut-off of 1 year was used to split our patient group into two cohorts for comparison. At 1 year, the damage from radiotherapy to healthy tissue should have stabilized in the majority patients. Specific outcomes of interest included the short-term complication of pharyngo-cutaneous fistula formation (defined as a clinical fistula visible on the skin), the longer-term complication of swallowing difficulties necessitating dilatation and the 5-year overall survival, using the date of laryngectomy as the start point.

Statistical analyses were performed using SPSS® Statistics 20.0 (IBM, Armonk, NY). Overall survival was calculated using the Kaplan–Meier method and Cox–Mantel log-rank test for comparison. The chi-squared test or binary logistic regression analysis was used as appropriate for univariate analysis.

This study does not fall under the scope of the Medical Research Involving Human Subjects Act and the institutional review board approved this study.

## Results

A total of 126 patients (108 males, 18 females) with a mean age of 66 years (range 44–87 years) underwent total laryngectomy after prior (chemo)radiotherapy in the study period. One hundred and two patients were operated as a salvage procedure due to recurrent cancer, 24/126 were operated for an afunctional larynx. Median follow-up following laryngectomy was 30 months (range 1–130 months). Overall 5-year survival following laryngectomy for the cohort was 35%. A total of 54 patients underwent laryngectomy within a year of their radiotherapy versus 72 patients who had a > 1-year time interval until laryngectomy. Univariate comparison of the cohort stratified by time interval of 1 year can be found in Table 1. No significant differences were found across a range of parameters between the two groups.

**Table 1:** Univariate comparison stratified by > 1-year time interval

	Time interval from end of radiotherapy to laryngectomy		
	< 1 year	> 1 year	*P* value
*N*	54	72	
Indication			
Salvage	45	57	
Functional	9	15	0.556
Gender			
Male	47	61	
Female	7	11	0.713
ASA			
1	25	28	
2	18	20	
3	10	19	
4	1	2	0.660
Age			
< 65	27	31	
> 65	27	41	0.439
Tumor site			
Supraglottis	8	19	
Glottis	23	35	
Subglottis	1	3	
Post cricoid	1	0	
Piriform sinus	12	8	
Transglottic	7	2	0.113
Previous Cetuximab			
No	50	67	
Yes	4	5	0.920
Previous cisplatinum			
No	45	65	
Yes	9	7	0.247
rT stage			
rT0 (functional)	9	15	
rT1	5	5	
rT2	16	18	
rT3	12	7	
rT4	12	27	0.181
rN stage			
rN0	46	60	
rN1	1	5	
rN2a	0	0	
rN2b	6	4	
rN2c	1	2	
rN3	1	1	0.440
Operation			
TL	35	41	
TLPP/TLP	19	31	0.372
Neck dissection			
None	33	40	
Unilateral	15	24	
Bilateral	6	8	0.790
Initial flap reconstruction			
None	33	41	
Pectoralis Major	20	23	
Radial forearm free flap	0	0	
Anterior lateral thigh	1	3	
Gastric pull-up	0	3	
Jejunum	0	2	0.414
Later dilatation			
No	42	59	
Yes	12	13	0.562
Pharyngo-cutaneous fistula			
No	36	48	
Yes	18	24	0.999

### Time delay to laryngectomy and overall survival

**Fig. 1:**
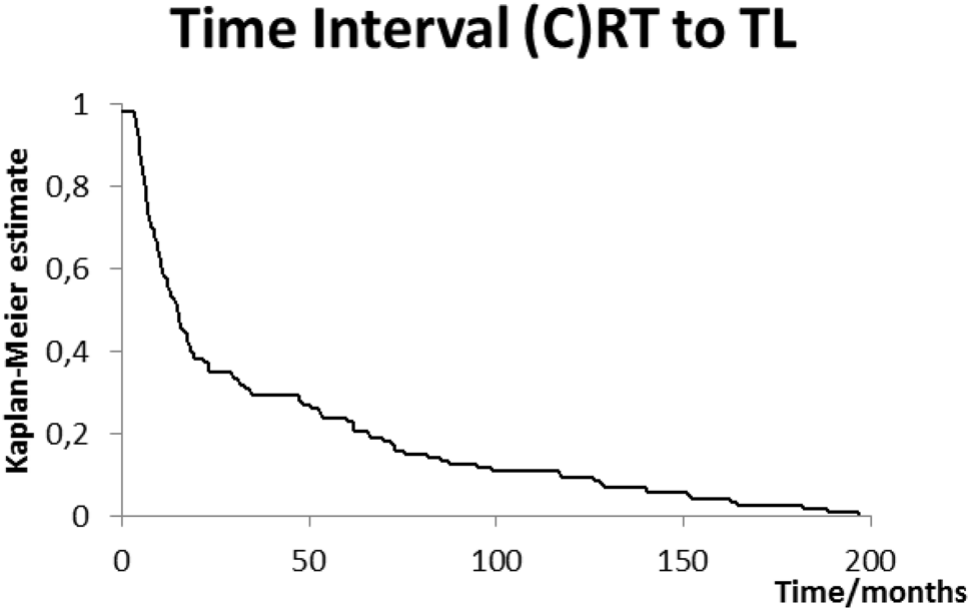
Time interval in months between end of radiotherapy and subsequent total laryngectomy (*n* = 126)

**Fig. 2:**
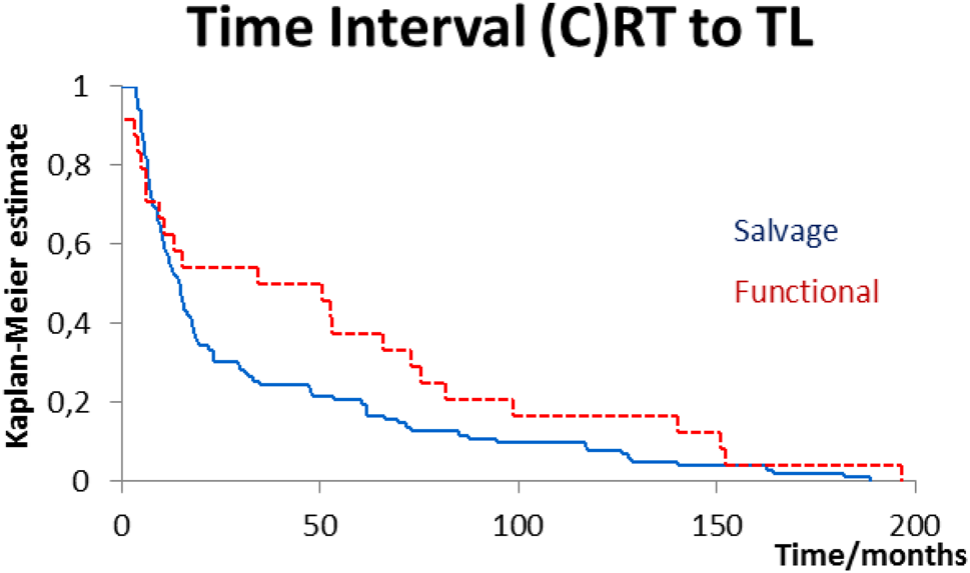
Time interval stratified for salvage (*n* = 102) vs functional (*n* = 24, *p* = 0.101)

Time interval between end of radiotherapy and laryngectomy is plotted in Fig. 1. The mean time interval was 36 months, the median interval 15 months (range 3–196 months). Figure 2 shows the different time delays stratified for treatment indication: median interval 5.3 months (salvage) versus 48 months (functional), *p* = 0.101.

**Fig. 3:**
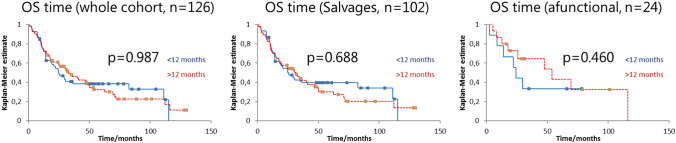
Overall survival for cohorts, stratified by < 1-year time interval vs > 1-year time interval

The 5-year survival was 38.5% (*n* = 54) for the < 1-year cohort versus 32.4% (*n* = 72) for the > 1-year interval cohort (*p* = 0.987, see Fig. 3). Once again, we stratified our cohort by treatment indication. For the salvage patients, 5-year survival was 35%, with the < 1-year cohort having a 5-year survival of 40% versus 30% for the > 1-year cohort (*p* = 0.688, see Fig. 3). For the functional laryngectomies, the 5-year survival was 39% (33% in the < 1-year cohort versus 40% in the > 1-year cohort, *p* = 0.46, see Fig. 3).

We also analyzed our patients with regard to whether the primary tumor was in the larynx or hypopharynx. For the whole cohort, the 5-year survival was 39% for laryngeal tumors vs 28% for hypopharyngeal, *p* = 0.244).

### Using alternative time interval measures

We further analyzed our cohort by splitting them at 6-month, 18-month, 24-month and 30-month intervals. The overall survival differences between the groups thus stratified remained statistically not significant (6 months *p* = 0.936, 18 months *p* = 0.955, 24 months *p* = 0.587, 30 months *p* = 0.407).

### Pharyngo-cutaneous fistula formation

A total of 42/126 (33%) patients developed a PCF postoperatively. When stratified according to time interval from radiotherapy to surgery, 18/54 (33%) of < 1-year interval patients developed a PCF versus 24/72 (33%) in the > 1-year cohort (*p* = 0.999). Once again, stratifying patients at time intervals of 6, 18, 24 and 30 months did not reveal any significant differences in risk of PCF formation. (6 months *p* = 0.631, 18 months *p* = 0.523, 24 months *p* = 0.597, 30 months *p* = 0.689).

### Risk of future dilatation

A later complication of total laryngectomy can be swallowing difficulties. Reported rates of such difficulties severe enough to require dilatation are roughly 23% [14]. In our cohort, 25/126 patients (20%) underwent dilatation, 12/54 (22%) in the short-interval cohort vs 13/72 (18%) in the long-interval cohort. On binominal logistic regression, this difference was not statistically significant, *p* = 0.562.

## Discussion

We present a single tertiary institution’s experience of laryngectomy in patients who have undergone previous (chemo)radiotherapy, specifically focusing on whether the time interval between end of (chemo)radiotherapy and laryngectomy is correlated with risk of PCF, need for dilatation and changes in overall survival.

Intuitively, it seems that the time interval to surgery would stratify patients in an informative way following (chemo)radiotherapy. In the salvage group, the time interval might imply radio-resistance on the part of the tumor. The distinction between residual and recurrent disease is debatable, but patients who were operated shortly after finishing their (chemo)radiotherapy might well have had more aggressive/advanced disease or at least more radio-resistant tumors than those only salvaged years later. Indeed, Weber et al. [22] argue that patients with a longer disease-free interval between initial treatment and recurrence are more likely to have a favorable outcome and that the finding of rapidly recurrent or persistent disease is predictive of poor outcomes, though this is not supported by our data. Note, however, that the most aggressive tumors presumably led to loco-regional irresectable disease or distant metastasis meaning that the patients were no longer candidates for salvage total laryngectomy and are not in our data set.

Furthermore, from a radiobiological point of view, it is clear that tissue quality after (chemo)radiotherapy is generally worse, particularly in a subset of patients who ultimately require functional laryngectomy. It might also be expected that this problem is particularly acute in patients operated soon after radiotherapy and that this would be reflected in increased rates of complications in both the salvage and functional groups.

The literature, however, contains conflicting evidence. A meta-analysis by Paydarfar et al. [19] found no association between time interval and PCF, though he does not mention survival outcomes. Van der Putten et al. [23] similarly showed no association between time interval and major complications, though it is worth noting that major complications here include all those that ‘‘needed surgery or admission to the intensive care unit or led to death’’ and the time interval used is not described.

In contrast, Basheeth et al. [18] and Scotton et al. [17] have shown that a short interval between radiotherapy and laryngectomy is a significant risk factor for the development of PCF. We could find no literature regarding the longer-term complication of swallowing difficulties requiring dilatation.

Our data show not only similar PCF and dilatation rates between the short- and long-interval patients, but also similar overall survival. It is also noteworthy that we describe 126 modern patients; this is 3 times [18] and 5 times [17] larger than single institution series discussed, without having the shortcomings of being a pooled analysis of data as in the DAHANCA [24] and American studies [19]. Furthermore, these 4 publications also relate to datasets dating as far back as the late 1970s when presumably radiotherapy techniques were less effective at sparing healthy tissue.

In other ways though, it seems our data are fairly consistent with these publications. Though they do not publish a figure similar to our Fig. 1, our median interval of 15 months compares with Grau et al.’s [24] 10 months and Scotton et al.’s 16.5 months. Paydarfar et al. and Basheeth et al. do not report median intervals. The latter, however, states that 30/47 (64%) patients underwent salvage laryngectomy within 1 year of (chemo)radiotherapy compared to our 54/126 (43%).

The incidences of PCF formation also seem similar in 3 of the 4 data sets with only Scotton et al.’s incidence of PCF as 58% being markedly higher. Our incidence is 33% compared with Basheeth et al.’s 34%, Payfardar et al.’s 25.7% and Grau et al.’s 30% (in the more modern patients). Only Grau et al. report a 5-year overall survival of 36% (vs our 35%), though not stratified for time interval.

Interestingly, the most recent analysis of time interval and PCF is a Dutch audit of 190 post (C)RT laryngectomies [21] which found that a *longer* interval between (chemo)radiotherapy and laryngectomy was a risk factor for PCF. Also, the cut-off used for short and long interval was 30 months (deemed as “clinically relevant”) which would stratify patients well into the chronic phase of inflammation and perhaps better delineate those with *ongoing* inflammation and those with a stable situation. When we analyze our data, however, with this 30 month cut-off, again we find no statistically significant differences in PCF rate (27/84 (32%) short-interval PCFs vs 15/42 (36%) long-interval PCFs, *p* = 0.689) or in overall survival (5y overall survival 35% vs 35%, *p* = 0.407).

### Flap use

Though not one of our primary aims in this investigation, no discussion of PCF can be complete without a mention of flaps. A total of 21/54 (39%) patients in the < 1-year cohort and 31/72 (43%) patients in the > 1-year cohort had a flap during their laryngectomy (see Table 1). Analysis of whether this reduced the PCF rate is unfortunately confounded by the fact that many of these flaps would have been used precisely in patients who were deemed at high risk of PCF. Furthermore, our data did not allow us to see whether in cases of PCF or flap failure, this was due to problems with the flap itself (for example, thrombosis) or problems with the in-setting (for example, dehiscence). It could be imagined that a healthy flap might well dehisce from an irradiated wound bed and that, therefore, a flap is not necessarily a panacea for PCFs.

## Conclusions

We present a modern, large, homogenous cohort of patients undergoing total laryngectomy after prior (chemo)radiotherapy. We find no association between the time interval between prior therapy and surgery and the risk of PCF formation, risk of dilatation or post operative overall survival.
